# Supplementary material for: Long‐lived macrophage reprogramming drives spike protein‐mediated inflammasome activation in COVID‐19
Source: EMBO Mol Med. 2021 Jun 16;13(8):e14150. doi: 10.15252/emmm.202114150 (PMC8350892; doi:10.15252/emmm.202114150)
Supplement: Supplementary file 1 — Appendix [file EMMM-13-e14150-s003.pdf]

Supplementary information for Theobald et al., "Long-lived macrophage reprogramming drives spike protein-mediated inflammasome activation in COVID-19"

**Appendix Figure S1 – S3**	**Additional data**	**Page 1-5**
**Appendix Table S1**	**Characteristics of blood donors for our study**	**Page 6-10**
**Appendix Table S2**	**Statistical data**	**Page 11-13**

Appendix Figure S1

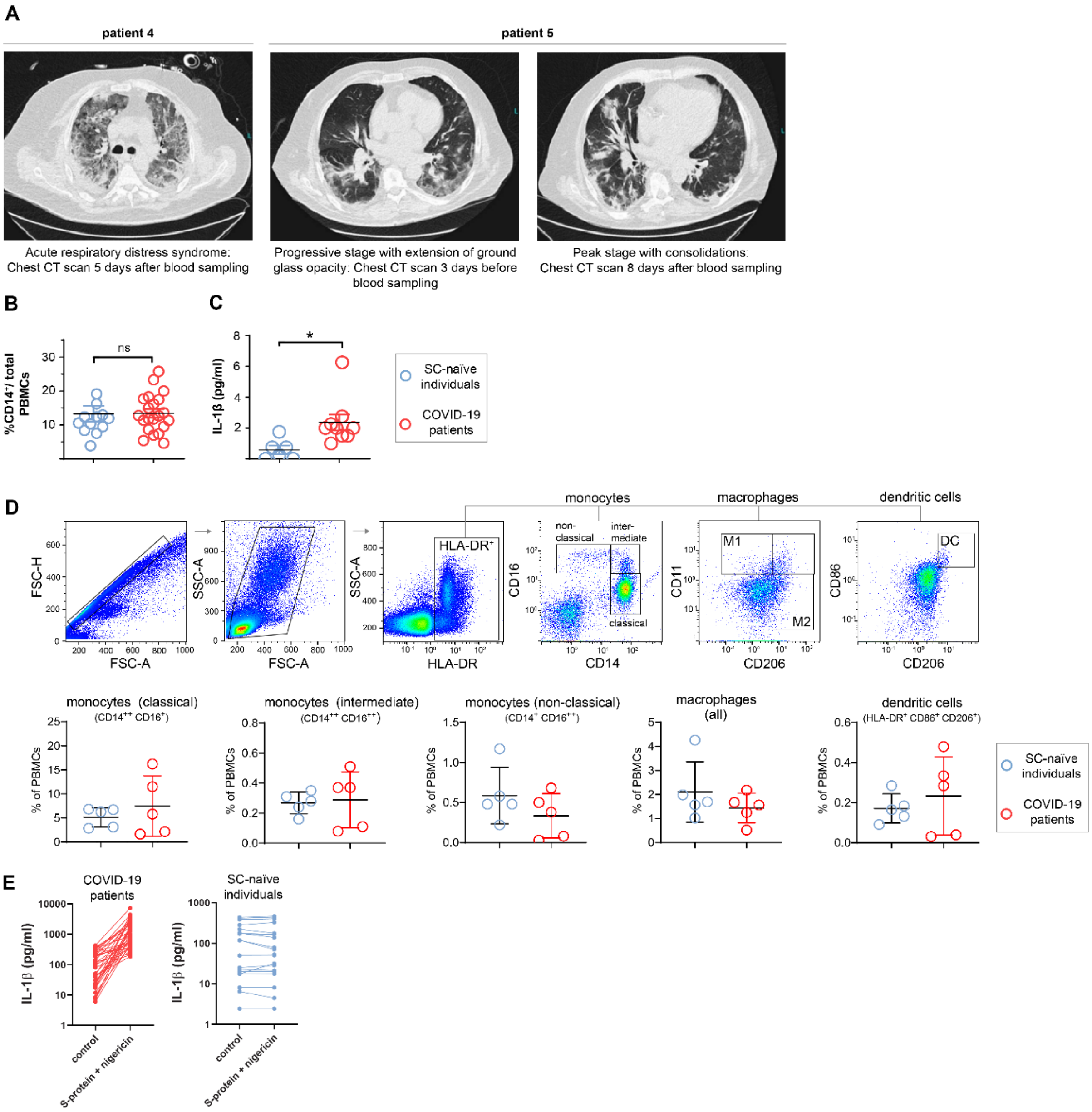

Appendix Figure S1: A Chest CT scan of patient 4 and 5 (see Supplementary tables 1) showing different pulmonal manifestations of disease. **B** Percentage of $CD14^{+}$ cells per number of total PBMC calculated after CD14 magnetic bead selection for healthy individuals (n=13, blue) and COVID-19 patients (n=18, red). Standard deviation of the mean is indicated. **C** Cytometric Bead Array-based quantification of IL-1β in plasma of COVID-19 patients (n = 6; red circles) or healthy individuals (n = 6; blue circles). For S1b and S1c students T-test with Welsh correction were performed and significances are indicated. **D** Representative gating strategy (top panel) showing flow cytometry analysis for PBMC phenotyping regarding monocytes (CD16, CD14), macrophages (CD11c, CD206) and dendritic cells (CD86, CD206). Quantifications (bottom panel, % of total PBMC) of classical monocytes ($CD14^{++}$/$CD16^{+}$), intermediate monocytes ($CD14^{++}$/$CD16^{++}$), non-classical monocytes ($CD14^{+}$/$CD16^{++}$), macrophages (M1+M1/2+M2, see gating example) and dendritic cells (HLA-DR^{+}/$CD86^{+}$/$CD206^{+}$) comparing healthy controls (blue) versus COVID-19 patients (red) (n=5 for healthy individuals and COVID-19 patients). **E** Quantification of IL-1β concentration (pg/ml) in the supernatants of primary macrophage cultures from COVID-19 patients (n = 44; red bars) or healthy individuals (SC-naïve; blue) after stimulation with S-protein (0.1 µg/ml) and nigericin subsequently. Control cells were left untreated. (log scale).

Appendix Figure S2

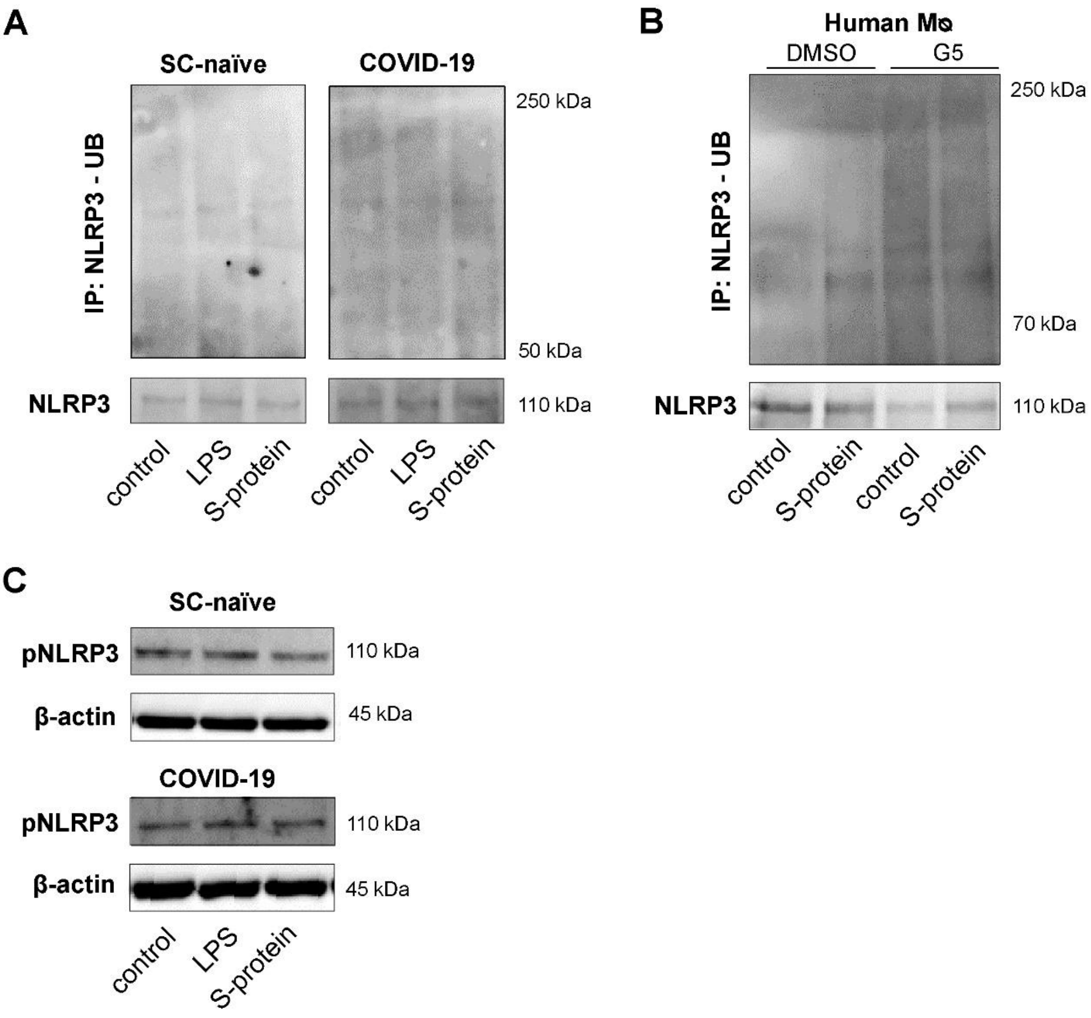

Appendix Figure S2: A Detection of upiquitinated NLRP3 in cell lysates from healthy (left) and COVID-19 (right) macrophages. Macrophages were stimulated with LPS or S-protein (4h) followed by 2h of nigericin treatment. Control cells were left unstimulated. NLRP3 (bottom) was detected in order to verify immune precipitation of the correct protein. Representative example of three individual experiments. **B** Detection of upiquitinated NLRP3 (top) total cell lysates from primary macrophages. Macrophages were stimulated with S-protein (4h) followed by 2h of nigericin treatment. 15min prior to nigericin the ubiquitinase isopaptidase inhibitor G5 (2µM) was added. Control cells were left unstimulated. NLRP3 (bottom) was detected in order to verify immune precipitation of the correct protein. Representative example of two individual experiments. Ubiquitin Antibody detects ubiquitin, polyubiquitin and ubiquitinated proteins (1:1000 dilution). **C** Detection of p-NLRP3 (110kDA, top row, antibody targets NLRP3 around the phosphorylation site of Ser295, dilution 1:1000) in total cell lysates

from healthy (upper panel) and COVID-19 (lower panel) macrophages. Macrophages were stimulated with LPS or S-protein (4h) followed by 2h of nigericin treatment. Control cells were left unstimulated and β-actin (45kDA) was used as loading control. Representative example of three individual experiments.

Appendix Figure S3

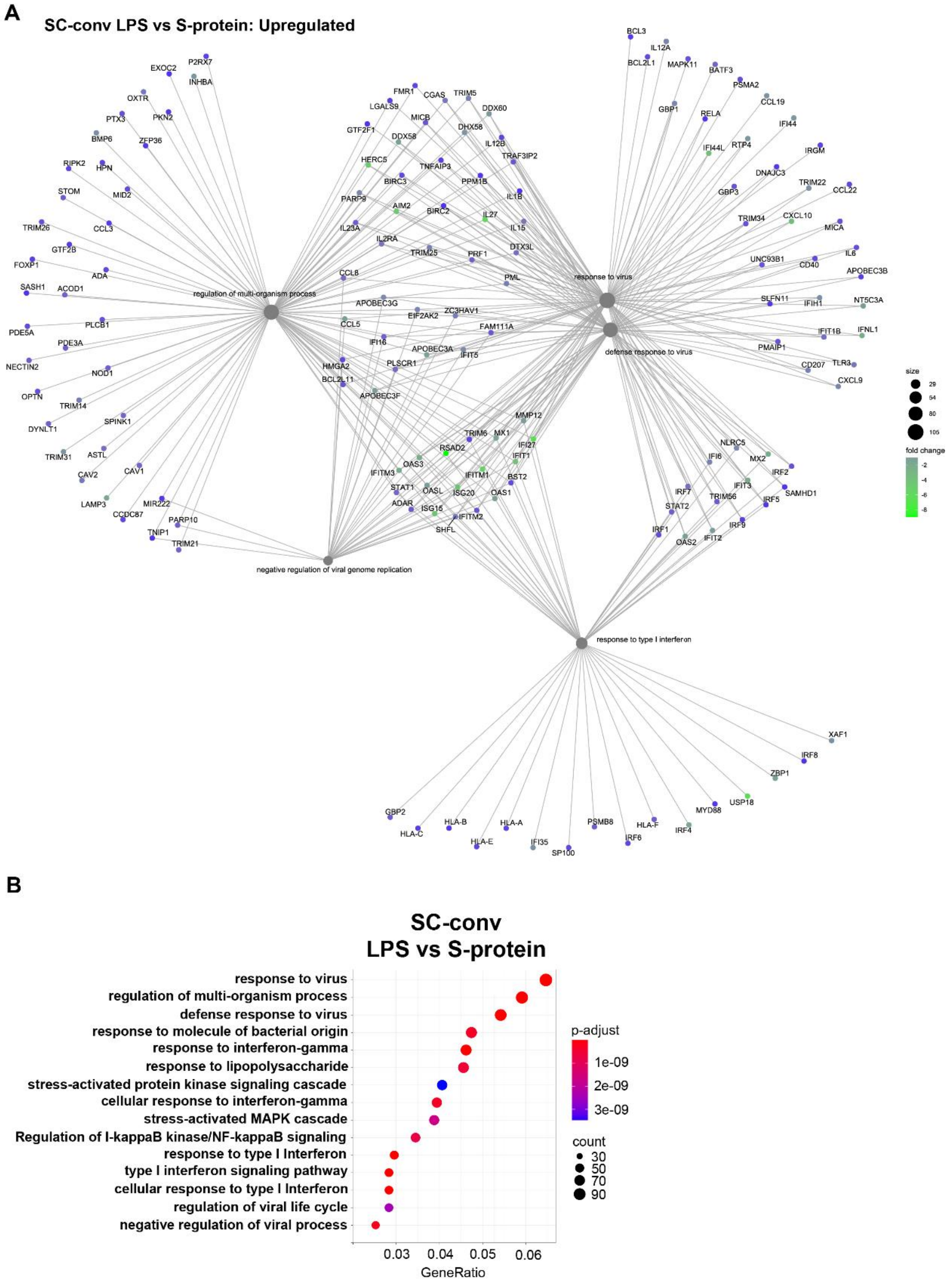

Appendix Figure S3: A Gene-concept network analysis, showing upregulated signaling pathways in LPS stimulated macrophages compared to S-protein stimulated macrophages in COVID-19 patient-derived cells (SC-conv). **B** Gene enrichment analysis indicating pathways activated in COVID-19 macrophages (SC-conv) stimulated LPS or S-Protein. Padj values are indicated (color code) and size of the circles corresponds to the number of DEGs (count). RNA-Seq analyses included data with padj <0.05 and $\log_2$-fold change of ≤-1 and ≥1.

Appendix Table S1: characteristics of blood donors for our study.

A

COVID-19 patients (severe disease/hospitalized)												
**SID**	**sex**	**age**	**O_2 therapy**	**comorbidites**	**sampling after dx (days)**	**Days of hospitalization**	**COVID-19 treatment**	**Antibiotic treatment**	**CRP (mg/ml)**	**ferritin (µg/ml)**	**ANC ($x10^9/l$)**	**ALC ($x10^9/l$)**
**1**	f	66	mask	myeloma	10	15	-	+	42	6585	1.4	0.4
**2**	m	52	mask	CKD, RA	6	12	-	+	95	3134	4.3	0.5
**3**	m	63	MV	HTN	18	22	-	+	126	1658	6.1	0.5
**4**	m	52	MV	none	15	43	-	+	247	6581	3.3	0.3
**5**	m	73	mask	AF, HTN, T2D	3	23	-	+	203	4542	2.5	0.5
**6**	m	57	mask	NASH	14	9	-	-	35	1901	3.6	1.6
**7**	f	50	mask	HIV, T2D	3	8	+[1]	-	85	656	4.3	0.9
**8**	f	45	mask	none	3	9	-	-	97	1352	5.2	1.1
**9**	f	54	MV	HTN	42	45	+[2]	+	16	N/A	2.1	1.6
**10**	m	69	mask	CLL, HTN	36	56	+[3]	+	3	N/A	9.7	0.6
**11**	f	75	mask	AF, HTN	9	12	+[4]	+	5	N/A	11.7	4.1
**12**	m	60	mask	NSCLC, AF	8	8	+[4]	+	252	7420	24.3	1.40
**13**	f	62	mask	HTN	4	7	+[4]	-	40	341	8.8	1.3
**14**	m	53	MV	CHD, HTN, T2D	14	25	+[4]	+	82	562	5.3	1.7
**15**	f	53	mask	HT	3	5	+[4]	-	18	403	3.2	1.6
**16**	m	79	mask	CKD, HTN, T2D	2	26	+[4]	+	91	483	4.8	1.0
**17**	m	77	mask	COPD, AF, CKD	17	14	+[4]	-	2.4	N/A	10.1	2.5
**18**	m	43	-	HIV	32	15	-	-	1.2	N/A	1.8	1.9

**19**	m	86	MV	CHD, HTN, HT	2	N/A	+[4]	+	27	226	11.2	1.1
**20**	m	77	mask	CKD, T2D, CHD	3	19	+[4]	+	142	916	3.0	0.4
**21**	m	57	mask	NSCLC, T2D	2	9	+[4]	-	32	1270	2.4	0.9
**22**	f	82	mask	COPD, HTN, CKD	5	6	+[4]	+	78	140	81	0.7
**23**	m	55	MV	DLBCL, HTN	6	26	+[4]	+	114	806	3.3	0.2

B

**COVID-19 patients (mild disease/outpatients) used for Fig. 3A**												
**SID**	**sex**	**age**	**O_2 therapy**	**comorbidites**	**sampling after dx (days)**	**Days of hospitalization**	**COVID-19 treatment**	**Antibiotic treatment**	**CRP (mg/ml)**	**ferritin (µg/ml)**	**ANC (x10^9/l)**	**ALC (x10^9/l)**
**24**	f	39	-	none	18	0	-	-	N/A	N/A	N/A	N/A
**25**	m	30	-	none	18	0	-	-	N/A	N/A	N/A	N/A
**26**	m	26	-	none	24	0	-	-	N/A	N/A	N/A	N/A
**27**	f	26	-	none	23	0	-	-	N/A	N/A	N/A	N/A
**28**	m	50	-	none	59	0	-	-	N/A	N/A	N/A	N/A
**29**	m	66	-	HTN	15	0	-	-	N/A	N/A	N/A	N/A
**30**	f	52	-	BA	61	0	-	-	N/A	N/A	N/A	N/A
**31**	m	19	-	T1D, BA	57	0	-	-	N/A	N/A	N/A	N/A
**32**	f	22	-	none	57	0	-	-	N/A	N/A	N/A	N/A
**33**	f	55	-	HTN	66	0	-	-	N/A	N/A	N/A	N/A
**34**	m	51	-	HTN, BA	65	0	-	-	N/A	N/A	N/A	N/A
**35**	m	28	-	none	20	0	-	-	N/A	N/A	N/A	N/A
**36**	m	28	-	none	22	0	-	-	N/A	N/A	N/A	N/A
**37**	m	24	-	none	28	0	-	-	N/A	N/A	N/A	N/A
**38**	m	23	-	none	18	0	-	-	N/A	N/A	N/A	N/A

**39**	m	29	-	none	30	0	-	-	N/A	N/A	N/A	N/A
**40**	f	27	-	none	30	0	-	-	N/A	N/A	N/A	N/A
**41**	f	18	-	none	32	0	-	-	N/A	N/A	N/A	N/A
**42**	f	18	-	none	32	0	-	-	N/A	N/A	N/A	N/A
**43**	f	31	-	none		35	-	-	N/A	N/A	N/A	N/A
**44**	m	41	-	none		27	-	-	N/A	N/A	N/A	N/A

C

**Convalescent COVID-19 patients (Gene expression analysis)**													
**SID**	**sex**	**age**	**O_2 therapy**	**comorbidites**	**sampling after dx (days)**	**Days of hospitalization**	**COVID-19 treatment**	**Antibiotic treatment**	**CRP (mg/ml)**	**ferritin (µg/ml)**	**ANC ($x10^9/l$)**	**ALC ($x10^9/l$)**	**SARS-CoV2 IgG**
**45**	m	67	-	HTN	24	0	-	-	0.6	134	2.18	1.70	+
**46**	m	21	-	none	32	0	-	-	7.7	327	4.94	3.14	+
**47**	f	34	-	none	49	0	-	-	0.6	66	2.28	1.65	+
**48**	m	31	-	none	40	0	-	-	0.6	50	3.62	2.62	+

D

Healthy individuals													
**SID**	**sex**	**age**		**comorbidites**					**CRP (mg/ml)**	**ferritin (µg/ml)**	**ANC ($x10^9$/l)**	**ALC ($x10^9$/l)**	**SARS-CoV2 IgG**
**49**	f	30		none					N/A	N/A	N/A	N/A	N/A
**50**	m	41		none					N/A	N/A	N/A	N/A	N/A
**51**	m	33		none					N/A	N/A	N/A	N/A	N/A
**52**	m	26		none					N/A	N/A	N/A	N/A	N/A
**53**	m	26		none					N/A	N/A	N/A	N/A	N/A
**54**	m	26		none					N/A	N/A	N/A	N/A	N/A
**55**	f	52*		BA					0.7	61	4.82	1.64	-
**56**	m	22*		none					1.4	138	2.74	2.09	-
**57**	f	62*		HTN					0.9	297	2.63	1.69	-
**58**	f	54		none					N/A	N/A	N/A	N/A	N/A
**59**	f	60		BA					N/A	N/A	N/A	N/A	N/A
**60**	f	47		none					N/A	N/A	N/A	N/A	N/A
**61**	m	49		none					N/A	N/A	N/A	N/A	N/A
**62**	m	45*		none					0.6	164	2.66	1.88	-
**63**	f	31		none					N/A	N/A	N/A	N/A	N/A
**64**	m	35		none					N/A	N/A	N/A	N/A	N/A
**65**	f	24		none					N/A	N/A	N/A	N/A	N/A
**66**	f	26		none					N/A	N/A	N/A	N/A	N/A
**67**	f	37		none					N/A	N/A	N/A	N/A	N/A

**68**	m	33		none					N/A	N/A	N/A	N/A	N/A
**69**	m	27		none					N/A	N/A	N/A	N/A	N/A
**70**	f	25		none					N/A	N/A	N/A	N/A	N/A
**71**	m	28		none					N/A	N/A	N/A	N/A	N/A
**72**	f	23		none					N/A	N/A	N/A	N/A	N/A

E

**Tuberculosis patients**										
**SID**	**sex**	**age**	**Organ involvement**	**sampling after dx (days)**	**Antibiotic treatment**	**CRP (mg/ml)**	**ferritin (µg/ml)**	**ANC ($x10^9/l$)**	**ALC ($x10^9/l$)**
**73**	m	38	lymph nodes (cervical, paratracheal), sternum	19/469	+	4.1/1.1	N/A	5.1/5.7	1.2/1.4
**74**	m	28	multiple bone lesions	4/551	+	47.6/0.6	N/A	5.9/4.4	1.3/1.7
**76**	m	25	peritoneum, lymph nodes (abdominal), ascites, iliitis, soft tissue	5/461	+	226/0.9	N/A	5.2/4.1	0.8/1.5
**77**	m	35	multiple lymph nodes (cervical, abdominal, mediastinum)	5/459	+	15.7/5.5	N/A	2.0/1.8	3.8/1.9
**78**	m	53	soft tissue, bones (multiple lesions)	9/371	+	69/15.1	N/A	6.8/7.1	0.8/2.3
**79**	m	50	pleura, lymph nodes	10/287	+	58.4/0.9	N/A	6.5/2.5	1/0.7

AF: atrial fibrillation; ANC: absolute neutrophil count; ALC: absolute lymphocyte count; BA: bronchial asthma; CHD: chronic heart disease; CKD: chronic kidney disease; CLL: chronic lymphocytic leukemia; COPD: chronic obstructive pulmonary disease; CRP: c-reactive protein; DLBCL: diffuse large B cell lymphoma; dx: diagnosis; HIV: human immunodeficiency virus infection; HT: hypothyroidism; HTN: hypertension; MV: mechanical ventilation; N/A: not available; NASH: Non-Alcoholic steatohepatitis; NSCLC: non-small cell lung carcinoma; RA: rheumatoid arthritis; T1D: Type 1 diabetes; T2D: Type 2 diabetes; + yes/positive; - none/negative; * Samples were used for gene expression analysis. [1]Tocolizumab/Placebo (Study); [2]Ribavirin/Lopinavir/Ritonavir; [3]Convalescent plasma therapy; [4]Remdesivir/Dexamethason

Appendix Table S2: Statistical data

Statistical data			
**Figure**	**Statistical test**	**Condition**	**p-value**
**1D**	Two-way ANOVA Tukey's multiple comparison test	Control SC-naïve vs LPS + nigericin SC-naïve	0.0050
		Control SC-naïve vs LPS + nigericin COVID-19	<0.0001
		Control SC-naïve vs S-protein + nigericin COVID-19	<0.0001
		LPS + nigericin SC-naïve vs LPS + nigericin COVID-19	0.0031
		S-protein + nigericin SC-naïve vs S-protein + nigericin COVID-19	<0.0001
**1E**	Two-way ANOVA with Tukey's multiple comparison test	Control SC-naïve vs LPS SC-naïve	<0.0001
		Control COVID-19 vs LPS COVID-19	<0.0001
		Control SC-naïve vs LPS + nigericin SC-naïve	<0.0001
		Control COVID-19 vs LPS + nigericin COVID-19	<0.0001
		Control SC-naïve vs S-protein SC-naïve	0.0029
		Control COVID-19 vs S-protein COVID-19	0.0023
		Control SC-naïve vs S-protein + nigericin SC-naïve	0.0015
		Control COVID-19 vs S-protein + nigericin COVID-19	0.0019
**2A**	One-way ANOVA with Tukey's multiple comparison test	DMSO vs MCC950	<0.0001
		DMSO vs VX-765	0.0010
**2E**	Kolmogorov-Smirnov test	Control SC-naïve vs S-protein COVID-19	0.0079
		Control COVID-19 vs S-protein COVID-19	0.0159
		S-protein SC-naïve vs S-protein COVID-19	0.0357
**3C**	Unpaired, two tailed t-test	SC-naïve vs SC-conv	0.043
**4A**	Unpaired, two tailed t-test	SC-naïve vs SC-conv	0.0332
**4B**	One-way ANOVA	DMSO vs KINK-1	0.0037

		DMSO vs MMG-11	0.0005
**4C**	One-way ANOVA	DMSO vs KINK	0.0019
**4D**	Two-way ANOVA with Tukey's multiple comparison test	Unstimulated SC-naïve vs DMSO SC-naïve	0.0447
		Unstimulated COVID-19 vs DMSO COVID19	<0.0001
		DMSO COVID-19 vs KINK-1 COVID19	<0.0001
		DMSO COVID-19 vs MMG-11 COVID19	<0.0001
**4E**	One-way ANOVA	1 ng/ml vs 10 µg/ml	0.0228
**5E**	Brown-Forsythe and Welsh ANOVA test	Unstimulated vs LPS + nigericin	0.0415
		Unstimulated vs FCCP + nigericin	0.0179
		Unstimulated vs H_2O_2 + nigericin	0.0247
		S-protein + nigericin vs FCCP + nigericin	0.0190
		S-protein + nigericin vs H_2O_2 + nigericin	0.0252
**5E**	Two-way ANOVA with Tukey's multiple comparison test	Control healthy vs LPS healthy	
		Control acute tuberculosis vs LPS acute tuberculosis before Tx	0.0022
		Control acute tuberculosis vs LPS acute tuberculosis after Tx	0.0027
		Control acute tuberculosis vs S-protein acute tuberculosis before Tx	0.0011
		S-protein healthy vs S-protein acute tuberculosis before Tx	0.0305
**EV1A**	Unpaired, two tailed t-test with Welsh correction	SC-naïve vs COVID19	0.0351
**EV1C**	Two-way ANOVA with Tukey's multiple comparison test	ATP COVID-19 vs LPS + ATP COVID-19	0.0017
**EV1D**	One-way ANOVA	DMSO vs MCC950	<0.0001
		DMSO vs VX-765	0.0003
**EV1F**	Two-way ANOVA with Tukey's multiple comparison test	Control SC-naïve vs LPS SC-naïve	<0.0001
		Control SC-naïve vs S-protein SC-naïve	0.0002
		Control COVID-19 vs LPS COVID-19	<0.0001

		Control COVID-19 vs S-protein COVID-19	<0.0001
**EV1J**	One-way ANOVA	DMSO vs S-protein + nigericin	0.0198
**EV1K**	Two-way ANOVA with Tukey’s multiple comparison test	S-protein COVID-19 vs S2-domain COVID-19	<0.0001
