# Supplementary material for: Long‐lived macrophage reprogramming drives spike protein‐mediated inflammasome activation in COVID‐19
Source: EMBO Mol Med. 2021 Jun 16;13(8):e14150. doi: 10.15252/emmm.202114150 (PMC8350892; doi:10.15252/emmm.202114150)
Supplement: Supplementary file 2 — Expanded View Figures PDF [file EMMM-13-e14150-s001.pdf]

Expanded View Figures

Figure EV1. Macrophages from COVID-19 patients secrete IL-1β upon stimulation with Spike protein.

A IL-1β (pg/ml) secreted from macrophages derived from SC-naïve (n = 6, blue) and COVID-19 patients (n = 9, red), which were stimulated with 10 μg/ml S-Protein and Nigericin. Student's t-test with Welsh corrections was used to calculate statistical differences as indicated.

B Quantification of IL-1β concentration (pg/ml) in the supernatants of primary macrophage cultures from COVID-19 patients (n = 8; red bars) or healthy individuals (n = 4; blue bars) stimulated with nigericin (2 h, 5 μM) without any other prior stimulation.

C Primary macrophages from COVID-19 patients (red bars) were stimulated with (n = 11) or without S-protein (0.1 μg/ml) (n = 18) or LPS (5 μg/ml) (n = 18) for 4 h. Subsequently, ATP (5 mM; 2 h) was used as signal 2 to induce IL-1β secretion. As control macrophages of healthy individuals were used (n = 6; blue bars). For statistical analysis, two-way ANOVA with Tukey post hoc test was used.

D Chemical inhibition of IL-1β (pg/ml) incubating macrophages from COVID-19 patients (red) for 2 h with DMSO (n = 44), MMC950 (n = 31), or VX-765 (n = 8), followed by 4 h incubation with LPS and additional 2 h with nigericin. For statistical analysis, one-way ANOVA with Tukey post hoc test was used.

E Results of three independent Western blot analyses (Healthy n = 3; COVID-19 n = 3) as described in Fig 2C. Graph shows relative NLRP3 expression correlated to the β-actin control. ImageJ Software was used to quantify protein band density.

F Quantification of total IL-1β concentration (pg/ml) from cell lysates derived from healthy individuals (blue; n = 3) and COVID-19 patients (red; n = 8) using ELISA. Macrophages were stimulated with LPS (5 μg/ml) or S-protein (0.1 μg/ml) or left unstimulated (control). For statistical analysis, two-way ANOVA with Tukey post hoc test was used.

G Quantification of the relative expression of pro-IL-1β compared to β-actin in macrophages of SC-naïve (blue; n = 3) and COVID-19 patients (red; n = 3) by western blot. Macrophages were stimulated with LPS (5 μg/ml) or S-protein (0.1 μg/ml) or left unstimulated (control).

H Detection of NEK7 in total cell lysates from healthy/SC-naïve (upper panel) and COVID-19 (lower panel) macrophages. Macrophages were stimulated with LPS or S-protein (4 h) followed by 2 h of nigericin treatment. Control cells were left unstimulated and β-actin was used as loading control. Representative example of three individual experiments.

I Quantification of the relative expression of NEK7 compared to β-actin in macrophages of SC-naïve (blue; n = 3) and COVID-19 patients (red; n = 3) by Western blot.

J Percentage of dead differentiated macrophages (% dead cells) from COVID-19 patients (n = 6, red) determined with cytometry via 7-AAD staining. Cells were stimulated with LPS or S-protein for 4 h and consequently for 2 h with nigericin. One-way ANOVA statistical text to calculate significances as indicated.

K IL-1β (pg/ml) secreted from COVID-19 patient-derived (n = 8; red bars) and SARS-CoV-2 naive (n = 5; blue bars) macrophages which were stimulated with full-length S-protein or S2-domain (all 0.1 μg/ml). Nigericin was used in both cases as signal 2. For statistical analysis, two-way ANOVA with Tukey post hoc test was used.

L Primary macrophages from COVID-19 patients (n = 8; red bars) or healthy individuals (n = 5; blue bars) were stimulated with ORF8 (0.1 μg/ml) for 4 h. Subsequently, nigericin (5 μM; 2 h) was used to induce IL-1β secretion.

Data information: Graphs show mean ± SEM. *P < 0.05; **P < 0.01; ***P < 0.001; ****P < 0.0001.

© 2021 The Authors

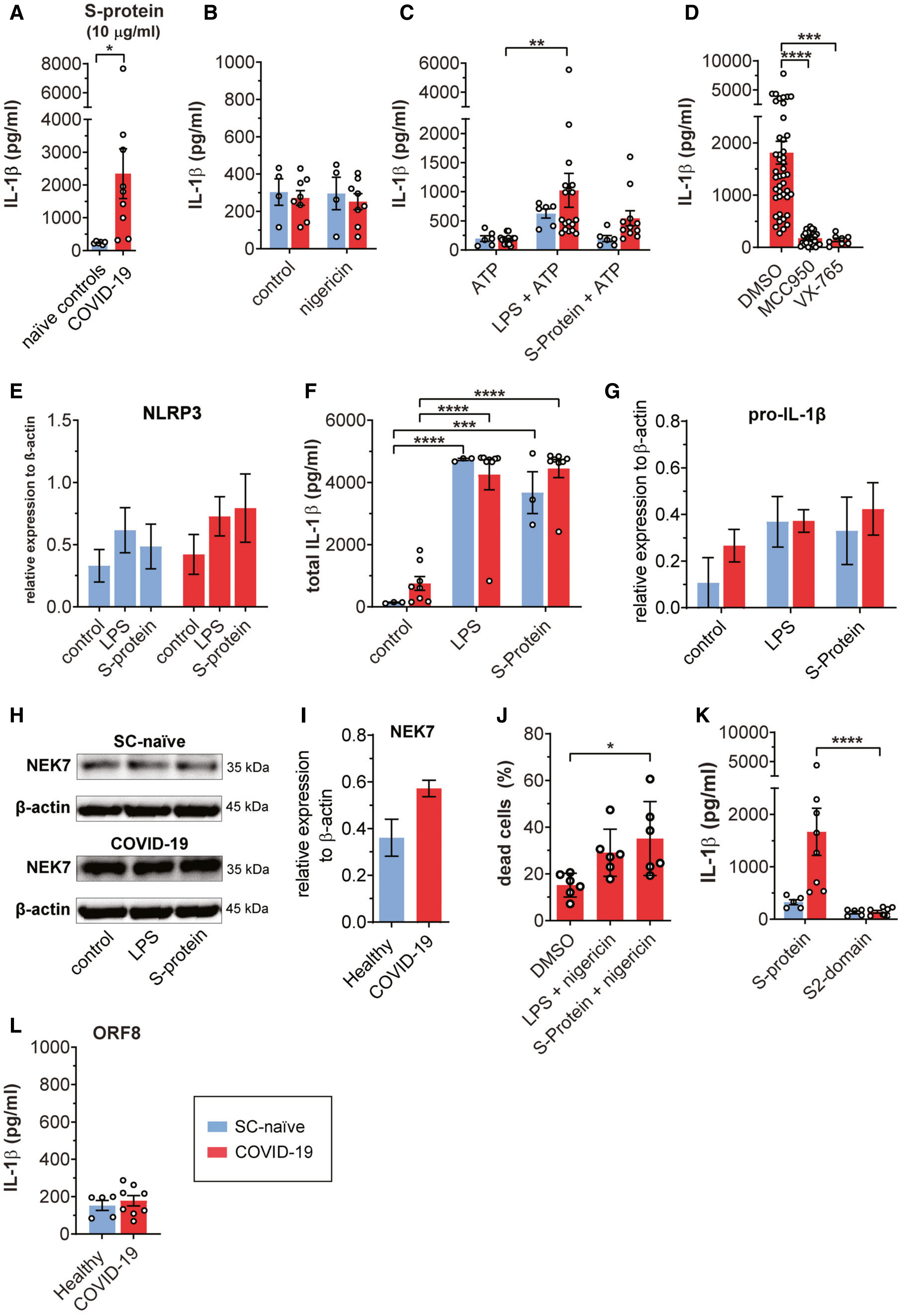

Figure EV1.

 © 2021 The Authors

Figure EV2. RNA-sequencing analysis comparing unstimulated and S-protein stimulated macrophages from SARS-CoV-2 naïve and COVID-19 patients.

A Principal component analysis (PCA) of macrophage RNA-Seq data indicating clustering of healthy (SC-naïve; blue) versus COVID-19 (SC-conv; red) groups when stimulated with S-protein, LPS, or without stimulation.

B Heat map indicating differential gene expression patterns comparing SC-conv macrophages stimulated with S-protein or left unstimulated. *Z*-score is indicated in a color score.

C Gene-concept network based on RNA-Seq data, showing upregulated signaling pathways in S-protein stimulated macrophages compared to unstimulated macrophages in COVID-19 patient-derived macrophages (SC-conv).

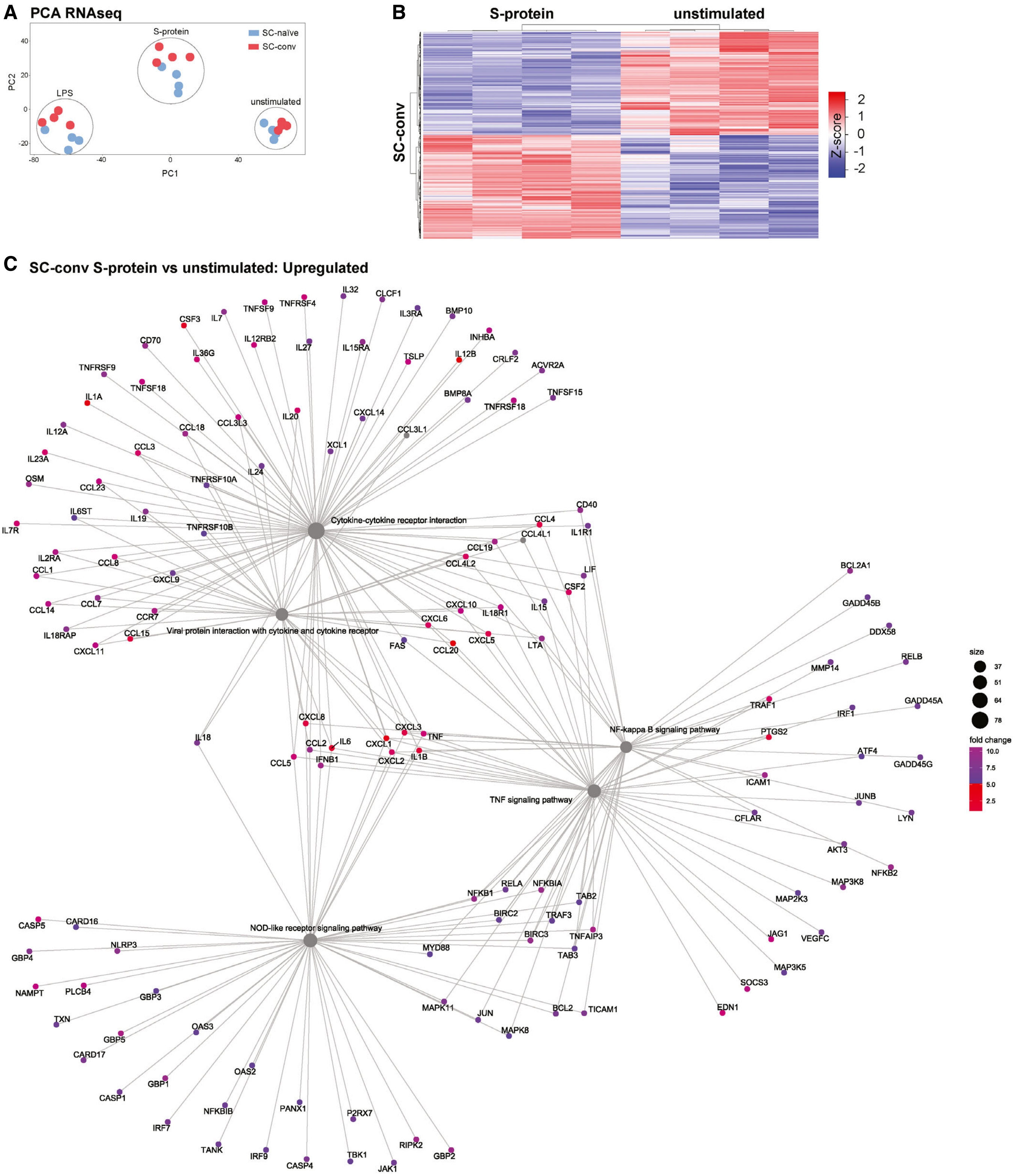

Figure EV2.

 © 2021 The Authors

Figure EV3. Clustering analysis of S-protein stimulated SARS-CoV-2 naïve and COVID-19 patient derived macrophages.

A Mean fluorescence intensity (MFI) of ACE2 expression on macrophages from healthy donors (blue; n = 3), COVID-19 (red; n = 3) detected by flow cytometry and compared to ACE2-negative cell line (n = 2).

B Venn diagram showing numbers of uniquely expressed genes in SC-conv macrophages stimulated with S-protein (red) compared to unstimulated macrophages (blue), deducted from DEGs found in SC-naïve macrophages stimulated with S-protein or left unstimulated. Size of the Venn diagram corresponded to the number of genes.

C Gene-concept network analysis, showing upregulated pathways in SC-conv versus SC-naïve macrophages stimulated with S-protein.

D Principal component analysis (PCA) from miRNA data indicating clustering from healthy (SC-naïve; blue) versus COVID-19 (SC-conv; red) macrophages without any stimulation. Each dot represents one individual (n = 4 for both experimental groups).

© 2021 The Authors

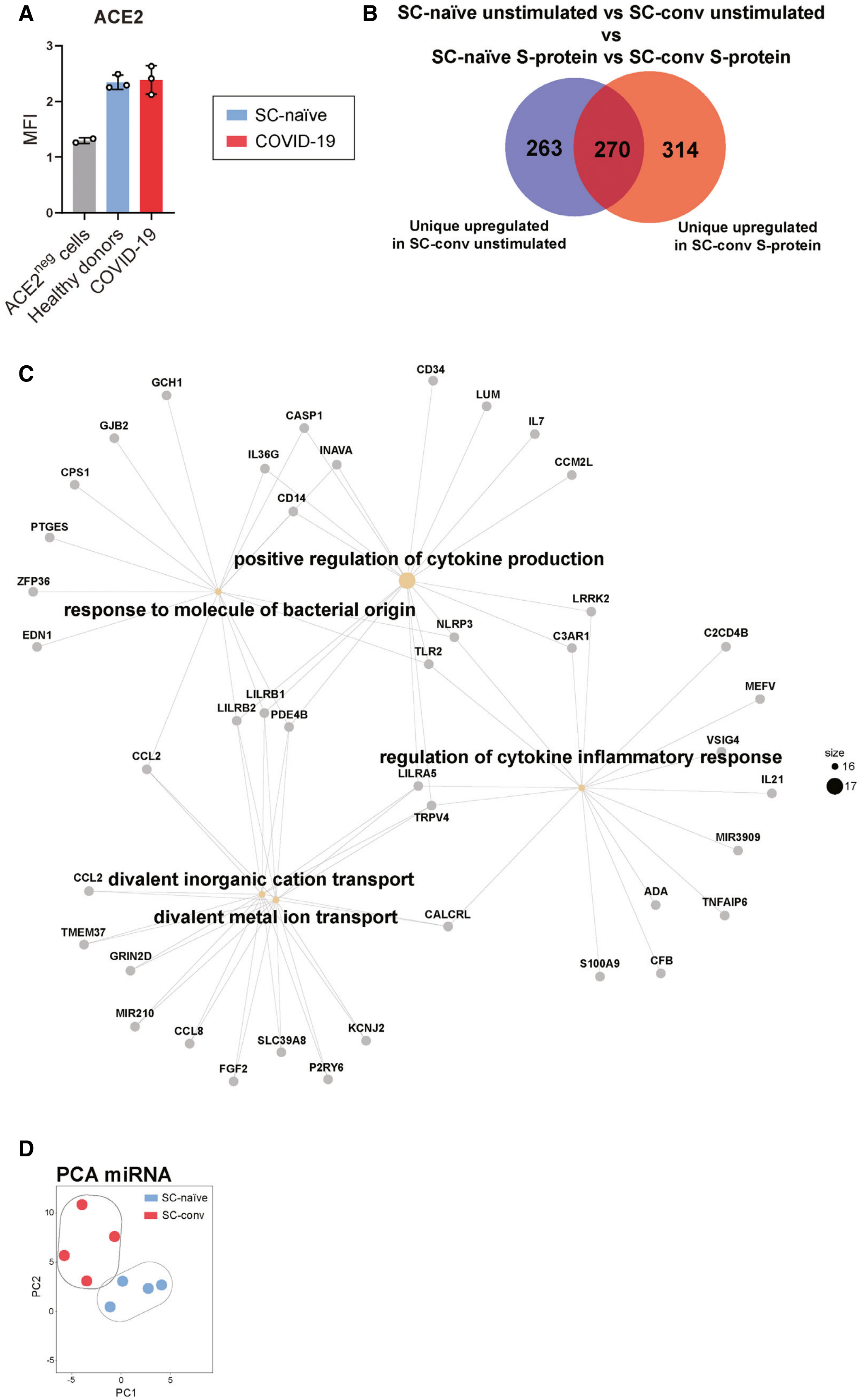

Figure EV3.

 © 2021 The Authors

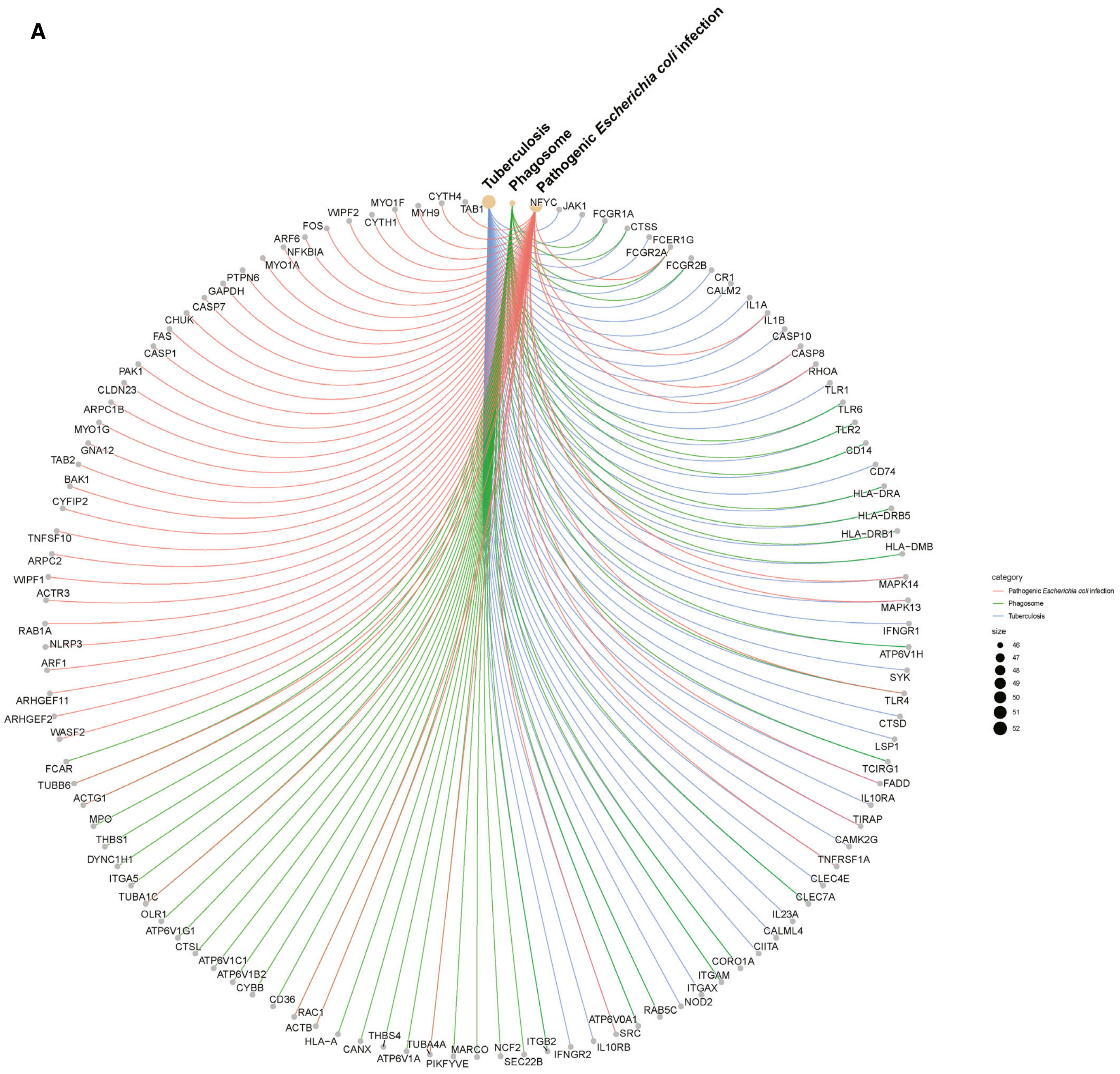

Figure EV4. CNET plot for differentially enriched loci of H3K27ac peaks identified in monocytes from convalescent patients.

CNET plot (derived from KEGG enrichment analysis shown in Fig 6D) illustrating genes involved in tuberculosis, *E. coli*infection, and phagosome KEGG pathways.

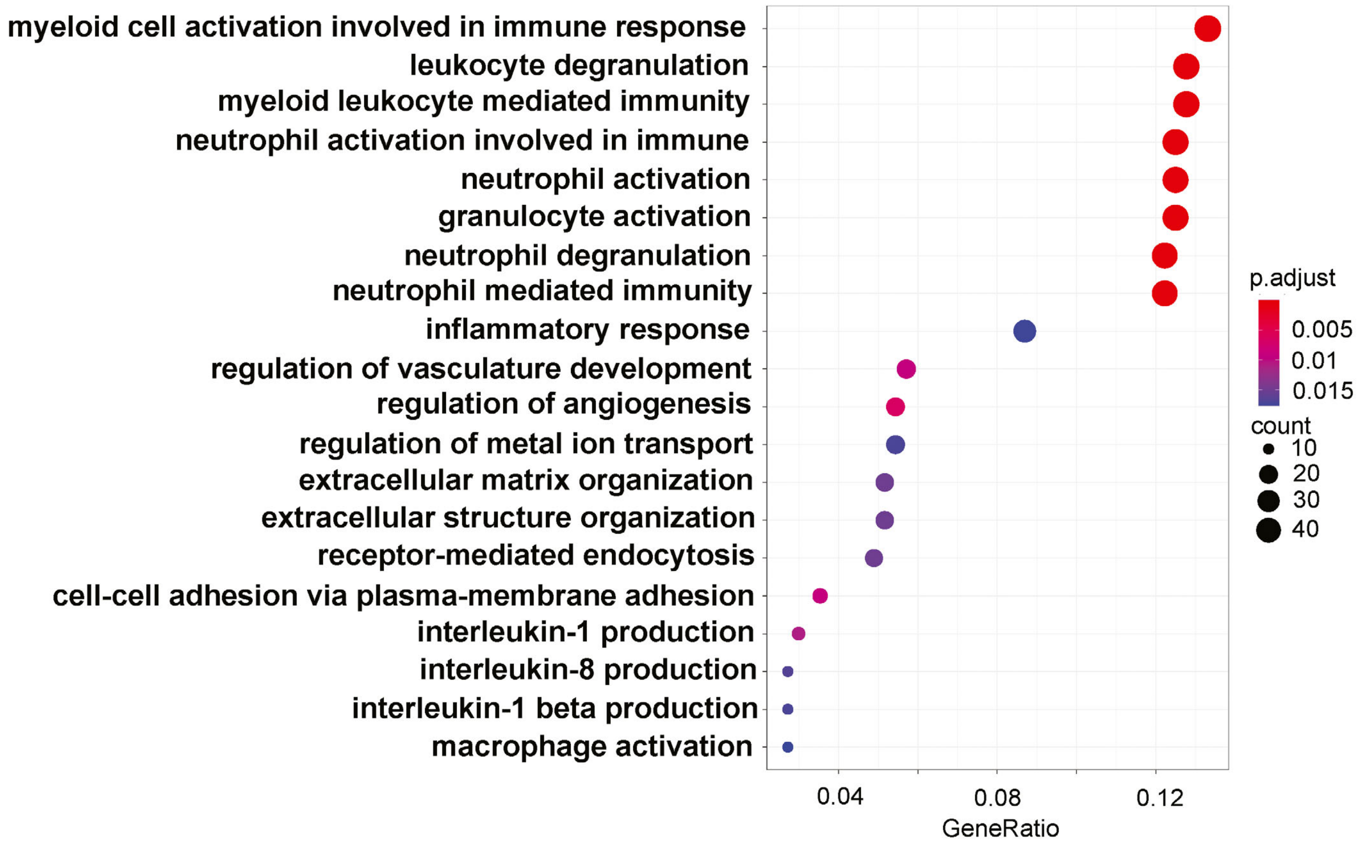

Figure EV5. GO analysis showing pathways activated in SC-conv monocytes.

GO pathway analysis performed on differentially enriched loci of H3K4me3 and H3K27ac-enriched peaks (promoters) showing associated pathways from SC-conv monocytes not present in SC-naïve monocytes. *P*-values were calculated by hypergeometric distribution, and the adjusted *P*-value was calculated with the Benjamini–Hochberg method.

 © 2021 The Authors
