# Supplementary material for: Long‐lived macrophage reprogramming drives spike protein‐mediated inflammasome activation in COVID‐19
Source: EMBO Mol Med. 2021 Jun 16;13(8):e14150. doi: 10.15252/emmm.202114150 (PMC8350892; doi:10.15252/emmm.202114150)
Supplement: Supplementary file 3 — Dataset EV1 [file EMMM-13-e14150-s004.zip › Legend.docx]

**Legend Dataset EV1:**

**Table A-H:** Results of differentially expressed genes (DEGs) analysis by RNA seq of macrophages of four fully recovered convalescent COVID-19 patients (SC-Conv) vs. four matched SARS-CoV-2 naïve controls (Naïve). Macrophages were stimulated with SARS-CoV-2 spike protein 1 µg/ml (SP), Lipopolysaccharide 5 µg/ml (LPS) for 4 h or left unstimulated (unstimulated).

**Table I:** Analysis of expression of miRNA in non-stimulated macrophages from four fully recovered convalescent COVID-19 patients (SC-Conv) vs. four matched SARS-CoV-2 naïve controls (Naïve).
