# Supplementary material for: Long‐lived macrophage reprogramming drives spike protein‐mediated inflammasome activation in COVID‐19
Source: EMBO Mol Med. 2021 Jun 16;13(8):e14150. doi: 10.15252/emmm.202114150 (PMC8350892; doi:10.15252/emmm.202114150)

Fig. 2B
NLRP3
β-actin
Western Blot

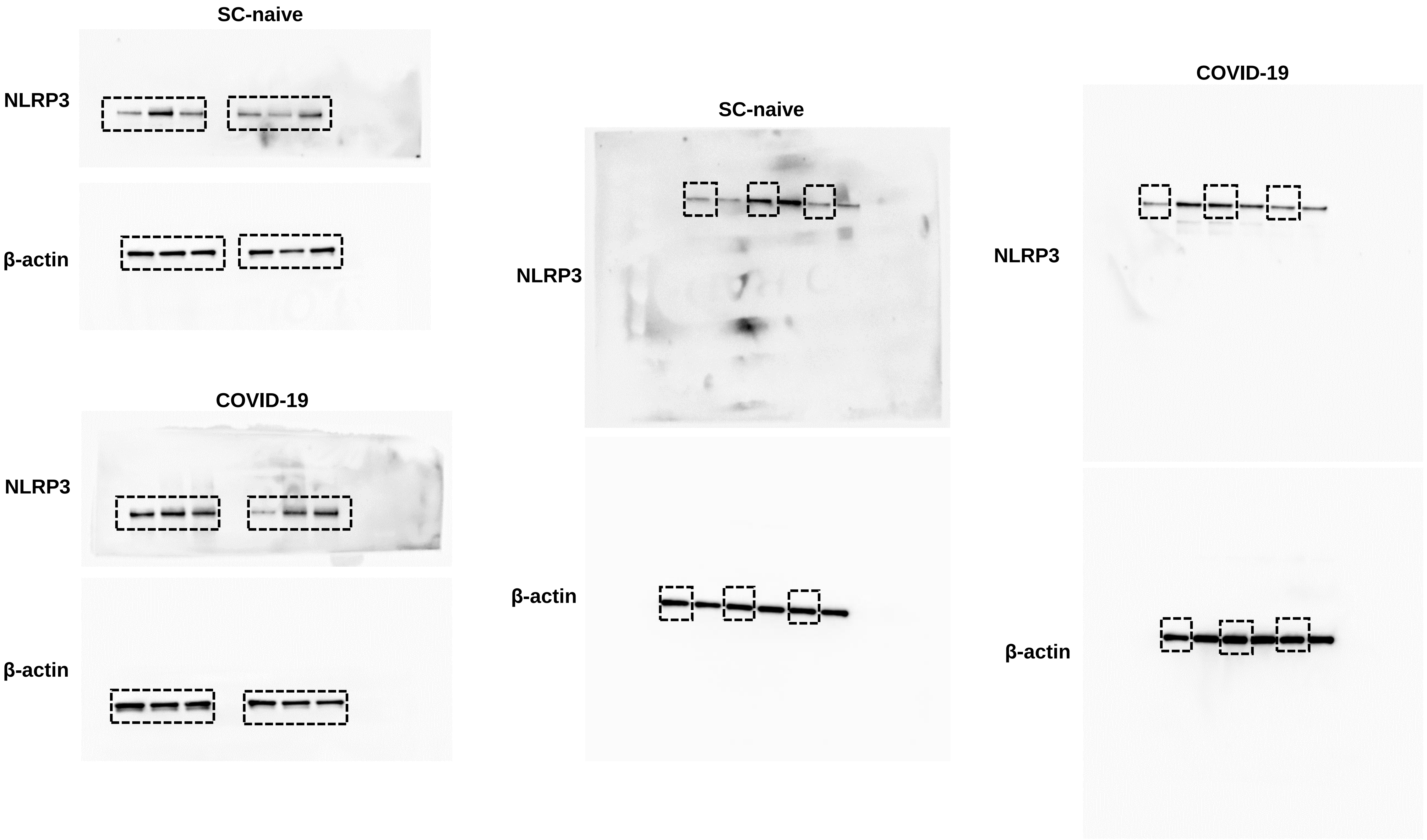

Fig. 2B
pro-IL-1β
β-actin
Western Blot

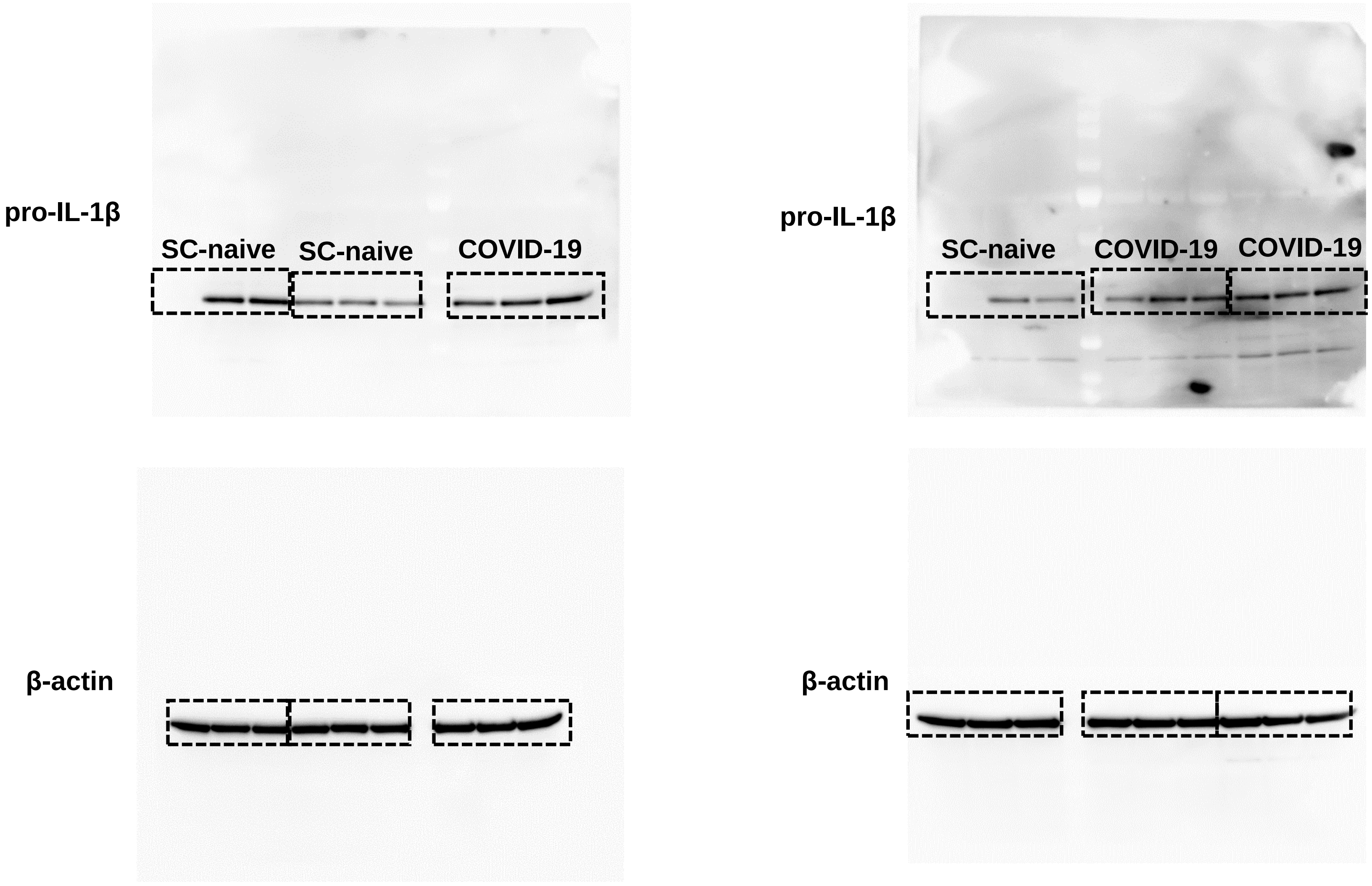

Fig. 2B
cIL-1β
β-actin
Western Blot

COVID-19
cIL-1β

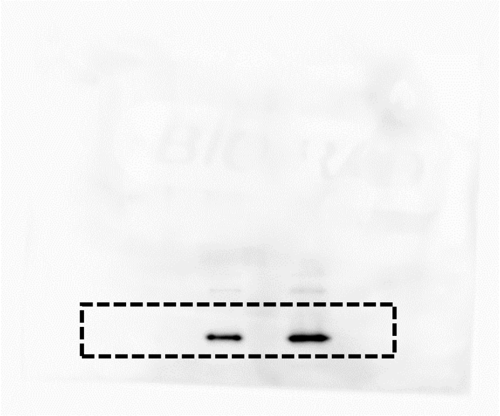

SC-naive
cIL-1β

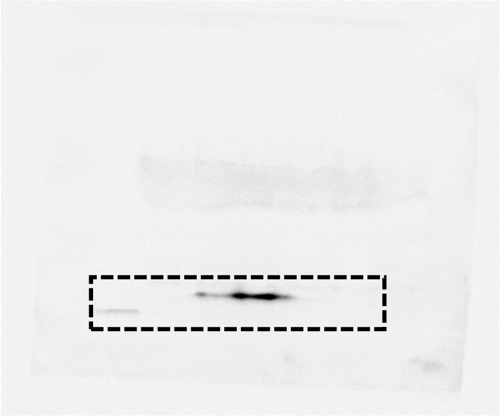

COVID-19
cIL-1β

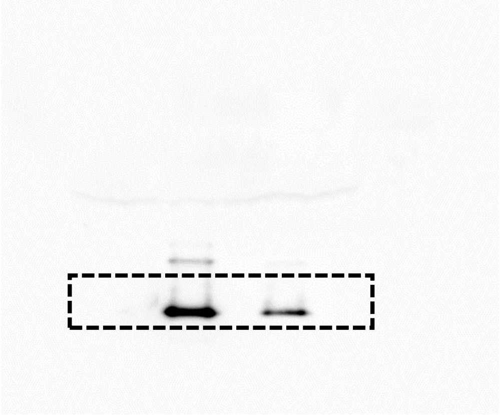

SC-naive
cIL-1β

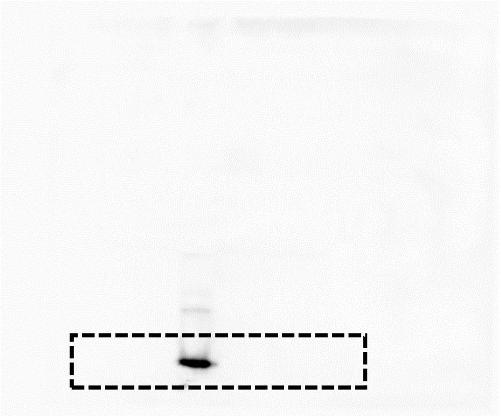

COVID-19
cIL-1β

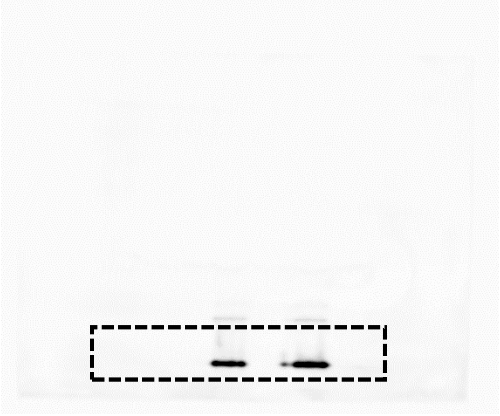

SC-naive
cIL-1β

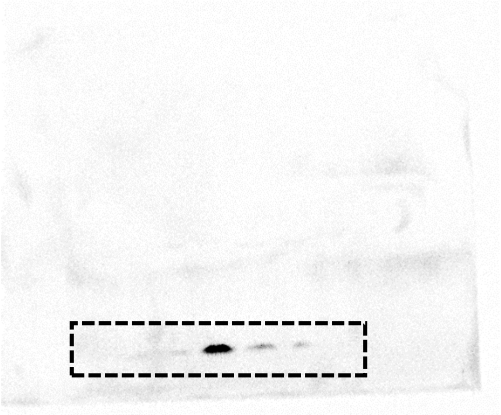

Fig. 2B
cGSDMD
β-actin
Western Blot

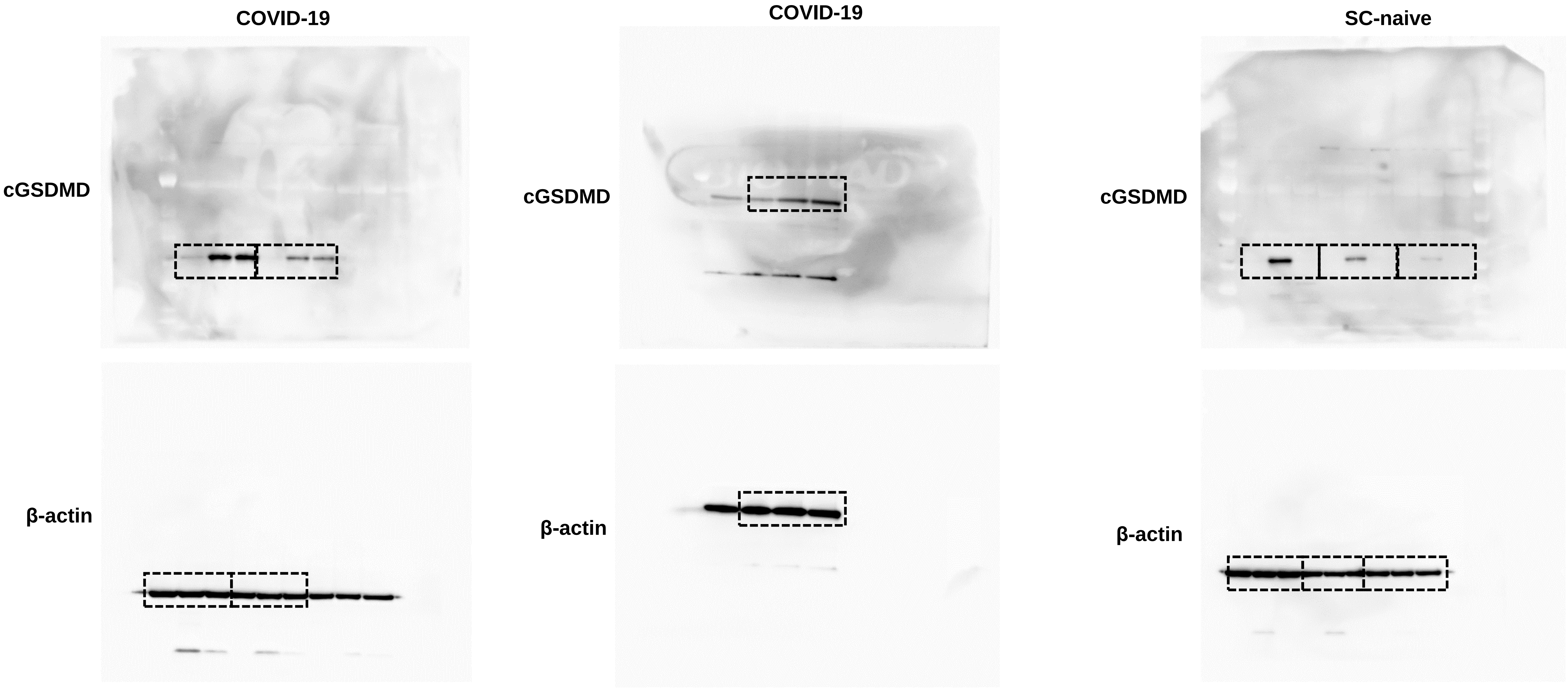

Supplement: Supplementary file 5 — Source Data for Figure 2 [file EMMM-13-e14150-s002.pdf]
